# The Effect of Polycystic Ovarian Syndrome on Development of Diabetes Mellitus by a Multistate Modelling Approach

**DOI:** 10.1002/edm2.70322

**Published:** 2026-08-27

**Authors:** Maryam Mousavi, Marzieh Saei Ghare Naz, Mahsa Noroozzadeh, Fereidoun Azizi, Fahimeh Ramezani Tehrani

**Affiliations:** ^1^ Reproductive Endocrinology Research Center, Research Institute for Endocrine Molecular Biology, Research Institute for Endocrine Sciences Shahid Beheshti University of Medical Sciences Tehran Iran; ^2^ Endocrine Research Center, Research Institute for Endocrine Disorders, Research Institute for Endocrine Sciences Shahid Beheshti University of Medical Sciences Tehran Iran; ^3^ Foundation for Research & Education Excellence Vestavia Hills Alabama USA

**Keywords:** diabetes, multistate model, polycystic ovarian syndrome, pre‐diabetes

## Abstract

**Background:**

Women with polycystic ovary syndrome (PCOS) are more susceptible to metabolic dysfunction, especially glucose impairment. Previous studies have considered prediabetes (pre‐DM) and diabetes mellitus (DM) as single‐point outcomes without accounting for the transition states. This study aimed to explore the transition between different blood glucose states using a multistate Markov model among women with and without PCOS.

**Methods:**

This study employed a dataset from the population‐based study of the Tehran Lipid and Glucose Study. Women, aged over 20 years, without pre‐DM and DM at baseline (1999–2000) and with documented PCOS status, were included; subsequent follow‐ups were conducted every 3 years, totaling six follow‐up assessments. Three states of ‘Normoglycemia’, ‘Pre‐DM’, and ‘DM’ and three possible transitions of (I) normoglycemia to pre‐DM, (II) normoglycemia to DM, and (III) pre‐DM to DM were defined. The multi‐state Markov models were applied to examine the predictive effect of PCOS on the transition rates throughout the progression of DM.

**Results:**

Out of 1587 participated women, 782 (49.3%) remained free of pre‐DM and DM at the end of the study. Transition from normoglycemia to pre‐DM was the most frequent progression observed, affecting 46.6% of the study participants. Women with PCOS significantly had 23% higher hazard of transition I [HR (95% CI): 1.23 (1.01–1.49); *p* = 0.034] compared to those without PCOS. While being PCOS did not significantly affect the risk of other transitions.

**Conclusions:**

This study underscores the significant impact of PCOS on the dynamic progression from normoglycemia to pre‐DM. Future longitudinal studies are essential to support our findings.

## Graphical abstract

PCOS was associated with a 23% higher risk of progression from normoglycemia → pre‐diabetes. PCOS was not significantly associated with direct progression of normoglycemia → DM or pre‐DM → DM.

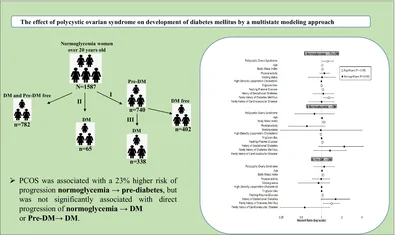

**Abbreviations**
ANOVA: one‐way analysis of variance
cholesterol HDL: high‐density lipoprotein
DM: diabetes mellitus
FPG: fasting plasma glucose
IQR: interquartile range
PCOS: polycystic ovary syndrome
pre‐DM: prediabetes
SD: standard deviation
TG: triglycerides
TLGS: Tehran lipid and glucose study

## 1 Introduction

Polycystic ovary syndrome (PCOS) is the most common endocrinopathy that affects 4%–20% of women of reproductive age worldwide [1]. Women with PCOS face elevated risks of infertility, obstetric issues, cardiovascular disease, metabolic disorders, and cancer [2]. PCOS is primarily a chronic condition with notable cardiometabolic effects [3], supported by genetic associations with diabetes mellitus (DM) and glycaemic traits [4]. DM per se is a multistage disease with long‐term complications [5]. Also, women in pre‐diabetes (pre‐DM) stage are at greater risk of transition to DM [6], particularly, higher fasting plasma glucose levels at baseline enhance this transition [7]. The progressive nature of these metabolic complications and understanding of how women with PCOS transition through different glycemic states is not clear.

In women with PCOS, a unifying mechanism of insulin resistance puts women at risk of developing impaired glucose tolerance [8]. Evidence reported a higher risk and incidence of diabetes and pre‐DM among women with PCOS [9, 10]. They also experience a higher rate of undiagnosed diabetes [11]. On the other hand, DM constitutes a significant global public health challenge, with its prevalence projected to increase markedly until at least 2045 [12, 13]. Notably, women are disproportionately affected by DM, experiencing more severe complications, higher morbidity rates, and greater adverse health outcomes compared to men [14]. A territory‐wide retrospective analysis in Hong Kong showed that women with PCOS are more likely to develop DM at an earlier age compared to healthy counterparts [15]. Abnormalities in insulin secretion, its signalling, and clearance have been observed among women with PCOS [16]. Hyperinsulinemia, insulin resistance, chronic inflammation, obesity, and hyperandrogenism may underlie the pathophysiology connecting PCOS and DM [17, 18]. However, the temporal patterns underlying the development and progression of DM remain incompletely understood.

Previous studies have considered the pre‐DM and DM as single‐point outcomes among women with PCOS, without accounting for the transition states [10, 19]. Traditional survival analysis approaches often overlook intermediate disease states, limiting their ability to accurately characterize the progression of complex conditions [20]. In contrast, the use of multistate models provides a more comprehensive framework for assessing disease progression by capturing transitions between distinct clinical states [21]. This approach enhances the understanding of the dynamic process from normoglycemia to pre‐DM and ultimately to DM, thereby facilitating earlier diagnosis and timely intervention. Applying multistate methodology allows for a robust evaluation of the role of PCOS in the development and progression of pre‐DM and DM.

The present study aimed to examine the influence of PCOS on the progression of glucose metabolism abnormalities, including pre‐DM and DM, utilizing data from a well‐defined cohort population study of the Tehran lipid and glucose study (TLGS).

## 2 Methods

### 2.1 Study Design and Setting

This study utilized a dataset of the TLGS. This prospective population‐based study was conducted to assess risk factors for non‐communicable diseases in a representative urban population of Tehran. The multi‐stage random cluster sampling method was used to select the 15,005 participants of TLGS. Previous studies explained the design and methodology of this cohort [22]; the baseline phase was conducted between 1999 and 2000. Subsequently, follow‐up assessments were performed at three‐year intervals, resulting in a total of six follow‐up visits.

For the present study, we included participants aged over 20 who had no prior medical diagnosis of pre‐DM and DM at baseline, whose status regarding PCOS was accurately documented. Among the 6493 women free of DM and pre‐DM at baseline phase, exclusions were applied as follows: participants younger than 20 years of age (*n* = 817), those without documented PCOS status (*n* = 1957), individuals exhibiting only isolated PCOS feature (*n* = 1327), participants with incomplete pre‐DM and DM data during follow‐up visits (*n* = 695), and those with missing information for covariates used in the adjustment models (*n* = 110). After these exclusions, a total of 1587 women remained eligible for the final analysis.

### 2.2 Data Collection

In each phase, data on demographic characteristics, obstetrics and gynaecological history, medical history, and family history were collected and recorded by trained staff members. Anthropometric measurements and blood pressure were also obtained following standardized protocols of TLGS.

Blood samples were taken between 7:00 and 9:00 AM, 12 h after overnight fasting, to measure fasting plasma glucose (FPG), high‐density lipoprotein (HDL), and triglycerides (TG) using the enzymatic colorimetric method [22, 23]. All intra‐ and inter‐assay coefficients of variation were less than 3.0%.

### 2.3 Definitions

Participants were classified into three glycemic categories based on their glucose profiles and medication history [7].

Type 2 diabetes was defined as having an FPG ≥ 126 mg/dL (≥ 7.0 mmol/L), a 2‐h post‐load plasma glucose ≥ 200 mg (≥ 11.1 mmol/L), or the current use of glucose‐lowering medications.

Prediabetes was identified in individuals not meeting the diabetes criteria who exhibited an FPG between 100 and 125 mg/dL (5.6–6.9 mmol/L) or a 2‐h post‐load plasma glucose between 140 and 199 mg/dL (7.8–11.0 mmol/L).

Finally, normoglycemia was defined as having an FPG < 100 mg/dL (< 5.6 mmol/L) or a 2‐h post‐load plasma glucose < 140 mg/dL (< 7.8 mmol/L) in the absence of glucose‐lowering medication or a history of diabetes.

PCOS was defined according to the Rotterdam criteria, requiring the presence of at least two of the following features [24, 25]: (1) Oligo/anovulation, (2) Hyperandrogenemia and/or hyperandrogenism, and (3) Polycystic ovaries.

Education level was categorized into two groups: < 12 years and ≥ 12 years of formal education. Current smokers were defined as individuals who reported smoking cigarettes or other tobacco products (such as water pipes or pipes) either daily or occasionally. Physical activity was assessed using the Modifiable Activity Questionnaire, with participants engaging in more than 600 min per week of metabolic equivalent tasks classified as having moderate to high physical activity [26].

### 2.4 Statistical Analysis

The baseline characteristics of the women who participated in this study are presented by the median and interquartile range (IQR) for non‐normally distributed continuous variables, and the mean and standard deviation (SD) for continuous variables that meet the normality assumption. To assess normality, we used the Kolmogorov–Smirnov test. To compare continuous variables between groups of DM status (final experienced event) we utilized one‐way analysis of variance (ANOVA) test or Kruskal‐Wallis test for variables with normal or skewed distribution in final event groups, respectively. Categorical variables are reported as frequencies (%) and compared between groups by Chi‐square $\left(\chi^{2}\right)$ test or the Fisher exact test (if there were cells with sparse data).

**FIGURE 1:**
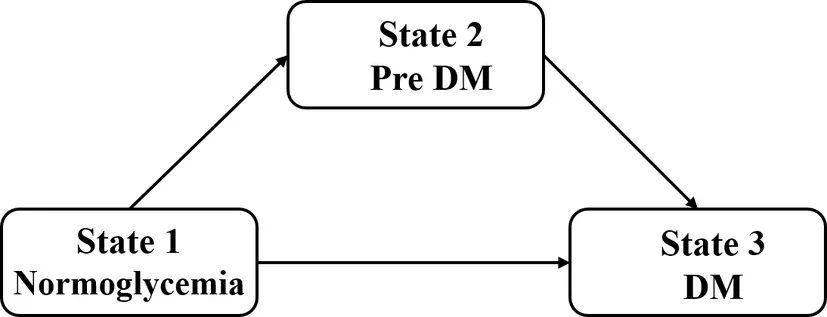
Theoretical states and transitions in the progression of glucose dysregulation. DM, diabetes; pre‐DM, pre‐diabetes.

We employed the Multi‐state Markov models to quantitatively assess the predictive impact of PCOS, the primary predictor of interest, on transition rates across different stages of DM progression. These models aim to estimate progression rates, evaluate the influence of individual risk factors, determine hazard ratios (HR) for transitions between disease complications, and provide predictive forecasts [27]. We assumed three states of ‘Normoglycemia’, ‘Pre‐DM’ and ‘DM’ and three possible transitions of (I) normoglycemia to pre‐DM, (II) normoglaicemia to DM, and (III) pre‐DM to DM. This assumed theoretical multi‐state model was considered in Figure 1.

According to the predefined theoretical model of study (Figure 1), participants begin in a healthy state (State 1), defined as being free of pre‐DM and DM. From this initial state, they may transition either to the pre‐DM (State 2) with a transition probability of q_12_ or directly to the DM state (State 3) with a corresponding transition probability of q_13_.

Participants who transition to the pre‐DM state (State 2) may subsequently progress to the DM state (State 3) with a transition probability of q_23_. Accordingly, this multi‐state model is represented by a 3‐by‐3 transition intensity matrix Q, which characterizes the rates of transitions between these states, as $Q=\left(\begin{array}{ccc} 0 & q_{12} & q_{13}\\ 0 & 0 & q_{23}\\ 0 & 0 & 0 \end{array}\right)$ and three possible transitions: (I) normoglycemia to pre‐DM, (II) normoglycemia to DM, and (III) pre‐DM to DM. It should be noted that, for all participants and all states, only the first entry into each state is taken into account in the analysis. To estimate each transition hazard in a multi‐state model, we performed Cox's proportional hazard model. The hazard of each transition represents the instantaneous risk of transitioning from one state of the model to another, which is determined by the current time and individual characteristics.

The baseline variables, including age, Body mass index (BMI), marital status, Physical activity, smoking status, HDL‐C, TG, FPG, family history of DM, history of gestational diabetes mellitus (GDM), and family history of cardiovascular disease (CVD), were selected as our final model's traditional risk factors. All statistical analyses were performed using R software version 4.4.2 and the ‘survival’ and ‘mstate’ packages. *p*‐value < 0.05 was considered statistically significant for all statistical tests.

## 3 Results

**FIGURE 2:**
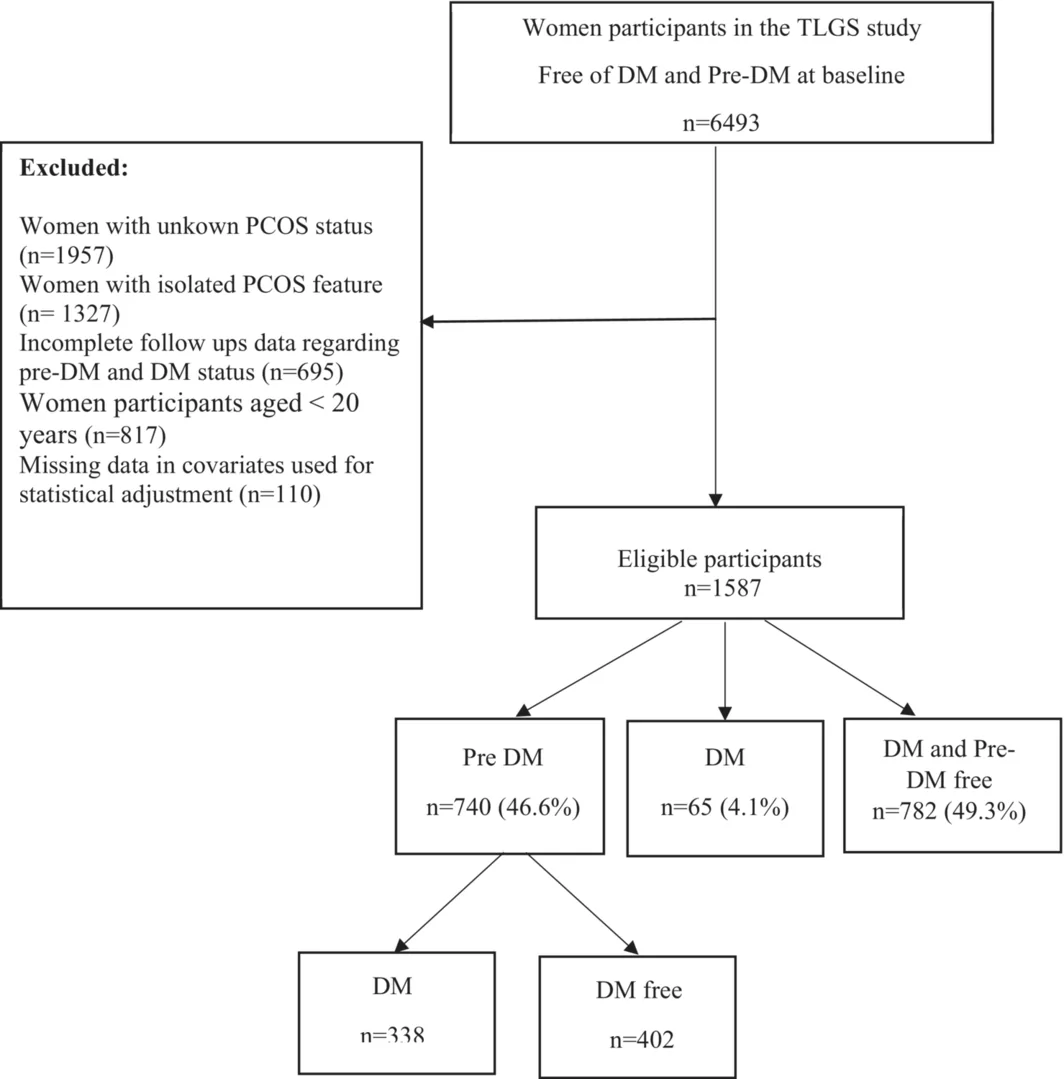
The study flowchart.

Figure 2 provides a detailed overview of the participants included and excluded from the study. The median (IQR) follow‐up time for pre‐DM and DM was 16.45 (11.05–19.60) years and 18.93 (15.55–20.19) years, respectively.

Table 1 presents the baseline characteristics of the study participants, both overall and stratified according to the most recent DM‐related event experienced. The median (IQR) baseline age of the study participants was 32 (26–38) years, and the mean (SD) of their BMI was 26.11 (4.61) kg/m^2^. Family history of DM and history of GDM were observed in 24.9% and 4.9% of study participants, respectively. Menopause was observed in 2.5% of the participants, and 30% of women had a moderate to high amount of physical activity. As shown in Table 1, individuals whose last experienced event was DM exhibited higher age and BMI, alongside a greater history of GDM and family history of DM (*p* < 0.001).

**TABLE 1:** Baseline characteristics of study participants overall and stratified by most recent DM status.

Variables		Total	Most recent DM status			
			Normoglycemia	Pre‐DM	DM	*p*
		*N* = 1587	*n* = 782	*n* = 402	*n* = 403	
Age (year)^b^		32 (26–38)	29 (25–36)	34 (28–40)	35 (29–41)	< 0.001
BMI (kg/m^2^)^a^		26.11 (4.61)	25.14 (4.34)	26.64 (4.32)	27.46 (4.97)	< 0.001
Educational level, years, *N* (%)	≤ Diploma	1377 (86.8)	663 (84.8)	359 (89.3)	355 (88.1)	0.062
	> Diploma	210 (13.2)	119 (15.2)	43 (10.7)	48 (11.9)	
Physical activity, *N* (%)	Low	1111 (70)	555 (71)	260 (64.7)	296 (73.4)	0.018
	Moderate to High	476 (30)	227 (29)	142 (35.3)	107 (26.6)	
Marital status, *N* (%)	Single	229 (14.4)	152 (19.4)	37 (9.2)	40 (9.9)	< 0.001
	Married	1358 (85.6)	630 (80.6)	365 (90.8)	363 (90.1)	
Smoking, *N* (%)	No	1542 (97.2)	761 (97.3)	388 (96.5)	393 (97.5)	0.651
	Yes	45 (2.8)	21 (2.7)	14 (3.5)	10 (2.5)	
Menopausal status, *N* (%)	No	1548 (97.5)	770 (98.5)	391 (97.3)	387 (96)	0.034
	Yes	39 (2.5)	12 (1.5)	11 (2.7)	16 (4)	
Family history of DM, *N* (%)	No	1192 (75.1)	623 (79.7)	292 (72.6)	277 (68.7)	< 0.001
	Yes	395 (24.9)	159 (20.3)	110 (27.4)	126 (31.3)	
History of GDM, *N* (%)	No	1509 (95.1)	753 (96.3)	380 (94.5)	376 (93.3)	0.066
	Yes	78 (4.9)	29 (3.7)	22 (5.5)	27 (6.7)	
Family history of CVD, *N* (%)	No	1538 (96.9)	758 (96.9)	388 (96.5)	392 (97.3)	0.826
	Yes	49 (3.1)	24 (3.1)	14 (3.5)	11 (2.7)	
HDL‐C (mg/dL)^b^		43.50 (39–53)	46 (39–53)	42 (35–49)	42 (35–48)	< 0.001
TG (mg/dL)^b^		103 (76–154)	90 (70–127)	119 (83.75–169)	128.50 (85.25–185)	< 0.001
FPG (mg/dL)^b^		86 (81–91)	84 (80–88)	87 (82–92)	89 (84–93)	< 0.001
PCOS, *N* (%)	No	1258 (79.3)	621 (79.4)	309 (76.9)	328 (81.4)	0.283
	Yes	329 (20.7)	161 (20.6)	93 (23.1)	75 (18.6)	

Abbreviations: BMI, body mass index; CVD, cardiovascular disease; DM, diabetes mellitus; FPG, fasting plasma glucose; GDM, gestational diabetes mellitus; HDL‐C, high‐density lipoprotein cholesterol; PCOS, polycystic ovary syndrome; pre‐DM, prediabetes; TG, triglyceride.

^a^ Mean (standard deviation).

^b^ Median (interquartile range).

**FIGURE 3:**
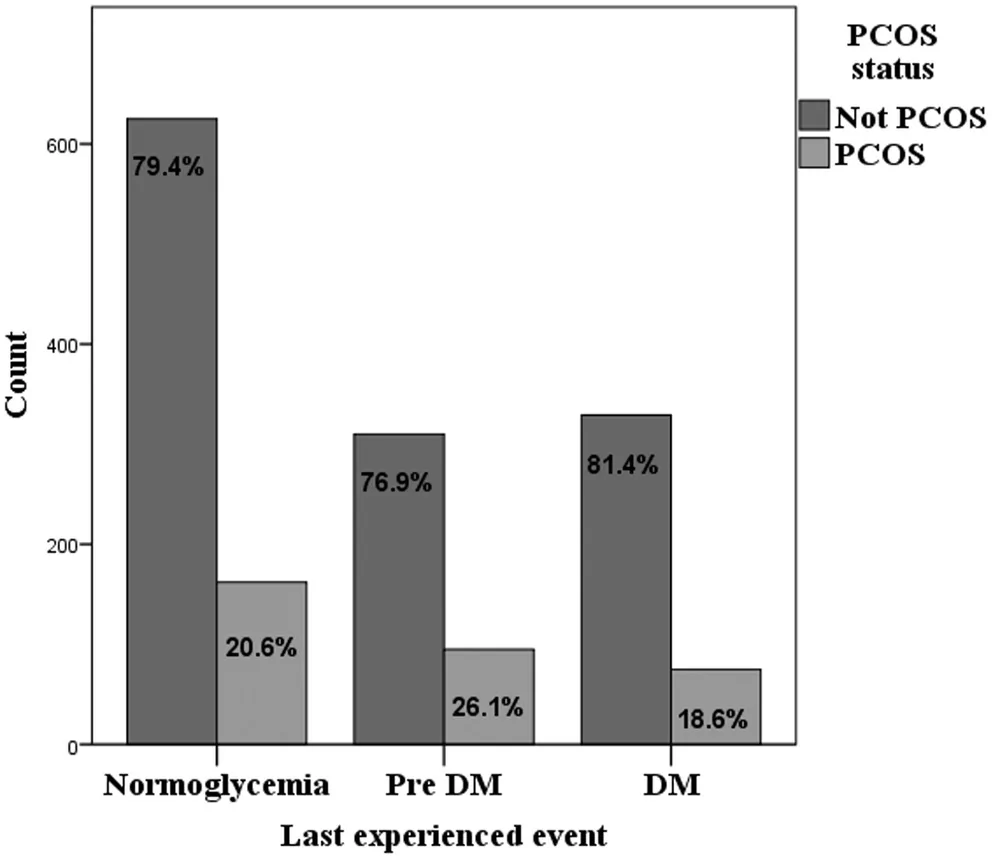
Distribution of individuals with PCOS categorized by their most recent diabetes status.

Figure 3 shows the frequency and percentage of individuals with and without PCOS in the last three participant statuses. As shown, 20.6%, 26.1%, and 18.6% of participants had PCOS status in normoglycemia, pre‐DM, and DM, respectively as the last experienced event in participants.

Table 2 shows the frequencies and transition probabilities of participants moving between model states throughout the study follow‐up period. As presented, all 1587 eligible participants at baseline were free of both DM and pre‐DM (state 1) and were therefore at risk of transitioning to pre‐DM (state 2) or DM (state 3). During follow‐up, 740 participants (46.6%) developed pre‐DM, 65 participants (4.1%) progressed directly to DM, while 782 participants (49.3%) remained free of both conditions until the end of the study. Among the 740 participants who transitioned to state 2 (pre‐DM), 338 individuals (46%) further progressed to state 3 (DM), whereas 402 participants (54%) remained in pre‐DM until the end of the study. Consequently, a total of 403 participants experienced state 3 during the follow‐up period.

**TABLE 2:** Frequency and probability of participant transitions between model states during the follow‐up period.

States	Total entering	Pre DM (%)	DM (%)	No event^a^ (%)
Normoglycemia, *N* (%)	1587	740 (46.6%)	65 (4.1%)	782 (49.3%)
Pre‐DM, *N* (%)	740	—	338 (46%)	402 (54%)
DM, *N* (%)	403	—	—	—

Abbreviations: DM, diabetes mellitus; pre‐DM, prediabetes.

^a^ No event: participants who did not experience the corresponding transition during the follow‐up period.

Table 3 shows the impact of PCOS as the primary independent variable on various transition stages of DM, highlighting the effects of different adjustment models.

**TABLE 3:** Impact of PCOS on DM progression across three transitions in a multistate model.

Variables at baseline	Transitions		
	I	II	III
	(Normoglycemia → pre‐DM)	(Normoglycemia → DM)	(Pre‐DM → DM)
	HR (95% CI)	HR (95% CI)	HR (95% CI)
	*p*	*p*	*p*
PCOS (ref: non‐PCOS)	**1.23 (1.01, 1.49)**	0.78 (0.40, 1.53)	1.09 (0.76, 1.54)
	***p* = 0.034**	*p* = 0.477	*p* = 0.626
Age (years)	**1.02 (1.01, 1.04)**	0.01 (0.96, 1.03)	1.01 (0.99, 1.03)
	***p* < 0.001**	*p* = 0.845	*p* = 0.064*
BMI (kg/m^2^)	**1.03 (1.01, 1.04)**	**1.08 (1.02, 1.14)**	**1.05 (1.01, 1.08)**
	***p* = 0.002**	***p* = 0.003**	***p* = 0.001**
Physical activity (ref: Low)	1.10 (0.93, 1.30)	0.60 (0.32, 1.12)	0.98 (0.72, 1.32)
	*p* = 0.256	*p* = 0.112	*p* = 0.898
Smoking status (ref: Non‐smoker)	1.01 (0.63, 1.63)	0.97 (0.23, 4.02)	0.90 (0.45, 1.32)
	*p* = 0.947	*p* = 0.967	*p* = 0.248
HDL‐C (mg/dL)	0.99 (0.98, 1.00)	**0.97 (0.94, 0.99)**	1.00 (0.99, 1.01)
	*p* = 0.282	***p* = 0.020**	*p* = 0.896
TG (mg/dL)	**1.00 (1.00, 1.01)**	1.00 (0.99, 1.00)	1.00 (0.99, 1.00)
	***p* < 0.001**	*p* = 0.058*	*p* = 0.752
FPG (mg/dL)	**1.05 (1.04, 1.06)**	**1.03 (1.01, 1.05)**	**1.04 (1.03, 1.06)**
	*p* **< 0.001**	*p* **< 0.001**	*p* **< 0.001**
History of GDM (ref: No)	1.06 (0.74, 1.51)	2.17 (0.90, 4.19)	1.62 (0.98, 2.65)
	*p* = 0.730	*p* = 0.081*	0.055*
Family history of DM (ref: No)	**1.27 (1.06, 1.51)**	1.33 (0.76, 2.32)	**1.40 (1.06, 1.86)**
	***p* = 0.007**	*p* = 0.307	***p* = 0.018**
Family history of CVD (ref: No)	1.00 (0.65, 1.53)	0.56 (0.44, 2.63)	0.56 (0.24, 1.30)
	*p* = 0.989	*p* = 0.316	*p* = 0.289

*Note:* Bold values show significance levels < 0.05.

Abbreviations: BMI, body mass index; CI, confidence interval; CVD, cardiovascular disease; DM, diabetes mellitus; FPG, fasting plasma glucose; GDM, gestational diabetes mellitus; HDL‐C, high‐density lipoprotein cholesterol; HR, hazard ratio; PCOS, polycystic ovary syndrome; pre‐DM, prediabetes; TG, triglyceride.

* *p*‐values indicating borderline statistical significance (0.05 ≤ *p* < 0.10).

The analysis revealed that women with PCOS had a statistically significant 23% increased hazard of transition I compared to those without PCOS (*p* = 0.034), whereas no significant differences in hazard were observed between PCOS and non‐PCOS participants for transitions II and III. Each additional year of age was associated with a statistically significant 2% increase in the risk of transition I (*p* < 0.001). Additionally, each unit increase in BMI was associated with a 3%, 8%, and 5% higher hazard of transitions I, II, and III, respectively (*p* < 0.05).

A family history of DM was significantly associated with increased transition risks of transitions I and III by 27% and 40%, respectively (*p* < 0.05).

Higher HDL‐C was associated with a 3% lower hazard of direct progression from normoglycemia to DM (*p* = 0.020), whereas higher TG was associated with an increased hazard of progression from normoglycemia to pre‐DM (*p* < 0.001).

Higher baseline FPG was consistently associated with increased hazards across all three transitions (Table 3).

**FIGURE 4:**
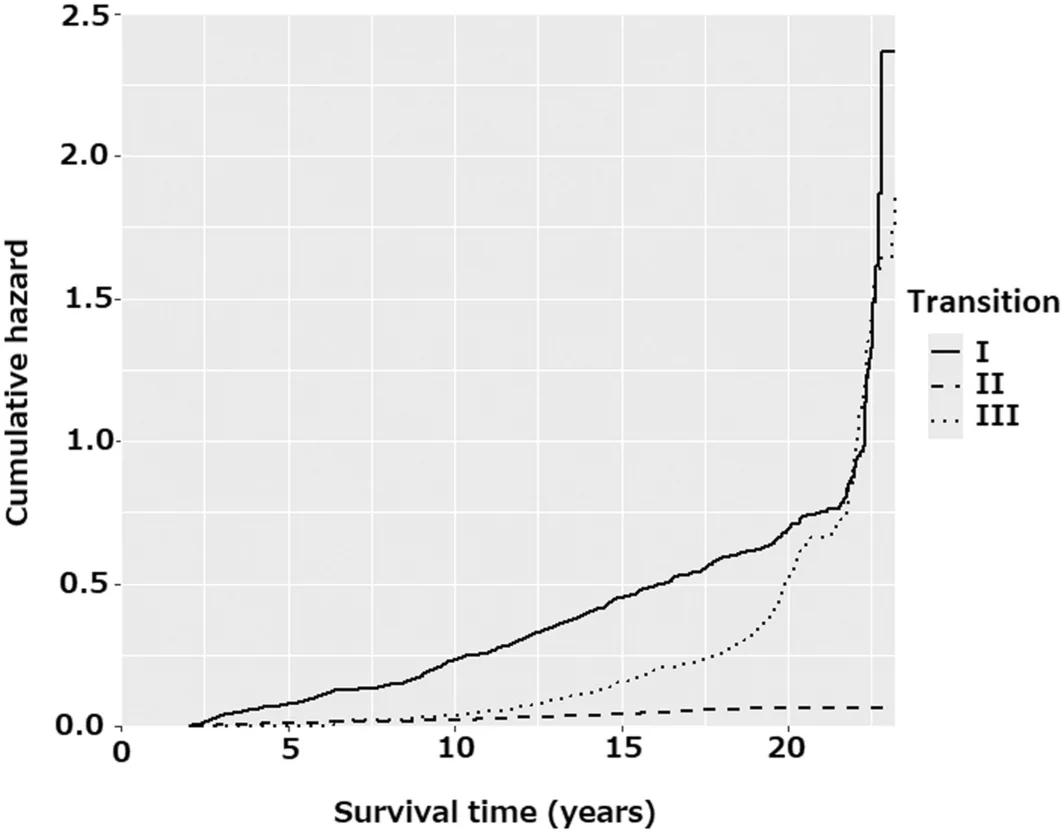
Cumulative hazard functions for different transitions of glucose impairments in a multistate model. I: Normoglycemia → pre‐DM, II: Normoglycemia → DM, and III: Pre‐DM to DM.

Figure 4 indicates the cumulative hazard functions of the three transitions within the multi‐state models. The greatest increase in hazard over time was observed in transition I, followed by transition III, whereas the cumulative hazard for transition II increased more steadily throughout the study period.

Table 4 shows the median and interquartile ranges of survival time in years in transitions of multistate models in total and between PCOS and control groups.

**TABLE 4:** Survival time for transitions in the multistate model in overall and stratified by PCOS status.

Group	Transitions survival time in years		
	Normoglycemia → pre‐DM	Normoglycemia → DM	Pre‐DM → DM
	Median (Q1–Q3)	Median (Q1–Q3)	Median (Q1–Q3)
Total	16.45 (11.05, 19.60)	18.77 (15.21, 20.15)	6.78 (3.17, 10.34)
Non‐PCOS	16.53 (11.02, 19.65)	18.75 (14.95, 20.16)	6.89 (3.15, 10.38)
PCOS	16.08 (11.19, 19.39)	18.80 (15.49, 20.13)	6.30 (3.467, 10.01)

Abbreviations: DM, diabetes mellitus; HR, hazard ratio; PCOS, polycystic ovary syndrome; pre‐DM, prediabetes.

## 4 Discussion

To our knowledge, this study represents the first investigation of the progression of glucose dysregulation across sequential glycemic states using multi‐state Markov models in women with and without PCOS. Our analysis revealed that women with PCOS exhibit a significantly elevated risk of progressing from normoglycemia to pre‐DM compared to their non‐PCOS counterparts. However, PCOS status did not significantly influence the subsequent transitions from pre‐DM to DM or direct progression to DM. Additionally, we identified that various risk factors including age, BMI, FPG, family history of DM, TG, and HDL‐C exert differential effects on these three distinct transitions.

Our findings revealed that patients with PCOS exhibited a significantly 23% higher hazard of transitioning from a healthy metabolic state to pre‐DM compared to individuals without PCOS. This elevated risk aligns with a wide range of previous investigations, including large‐scale cohort studies and reviews, which consistently demonstrate an increased prevalence and incidence of pre‐DM and DM among women with PCOS [9, 19, 28, 29]. However, these studies have largely treated pre‐DM and DM as isolated, single‐point outcomes, which limits the understanding of the temporal progression of metabolic dysfunction in women with PCOS. Our approach advances the existing body of literature by applying longitudinal analytical methods that evaluate transition probabilities between glycemic states over time. By modelling the sequential transitions between glycemic states of metabolic state changes, such as transitions from normoglycemia to pre‐DM and subsequently to diabetes, our study provides a more detailed depiction of how PCOS is associated with specific stages of glucose dysregulation.

Findings of our study failed to show the significant effect of PCOS on the transition from per‐DM to DM or normoglycemia to DM. Although women with PCOS are generally considered to be at increased risk for DM and its complications due to underlying insulin resistance and metabolic disturbances, a recent cohort study has reported that women with PCOS had a lower risk for diabetic complications than their healthy counterparts [30]. Several factors, including earlier diagnosis, closer clinical monitoring, and greater engagement in preventive lifestyle interventions among women with PCOS, may explain this paradox. It is widely proposed that lifestyle modification should be the first‐line treatment for women with PCOS [31], as it significantly improves both biochemical and clinical parameters such as insulin sensitivity, androgen levels, and ovulatory function. Moreover, LSM has been shown to reduce long‐term cardiometabolic risks, including the development of DM, hypertension, and cardiovascular disease in this population [32]. Through sustained adherence to tailored lifestyle modifications, women with PCOS can effectively mitigate the metabolic consequences associated with their condition, thereby lowering the incidence and severity of diabetic complications despite the higher baseline risk.

The association between PCOS and glucose metabolism abnormalities is primarily driven by insulin resistance, a hallmark feature of PCOS, which leads to compensatory hyperinsulinemia and subsequent impairment in glucose homeostasis. Additionally, factors such as chronic low‐grade inflammation, altered adipokine profiles, and androgen excess contribute to the pathophysiology linking PCOS and glucose dysregulation [33, 34]. Evidence supports that multiple genetic variants and loci involved in insulin secretion and signalling pathways contribute to the metabolic disturbances observed in women with PCOS [35, 36]. These genetic predispositions, combined with epigenetic modifications, play a critical role in the complex pathophysiology underlying PCOS [37]. Additionally, factors such as epigenetic changes, hyperandrogenemia, and obesity, which are commonly associated with PCOS, all contribute to insulin resistance. Hyperandrogenemia, in particular, worsens insulin sensitivity by interfering with insulin receptor signalling, while obesity amplifies metabolic dysfunction through increased adipose tissue‐derived inflammatory mediators and altered adipokine secretion [38]. Moreover, gut‐brain hormonal signalling, gut dysbiosis‐driven inflammation, and immune modulation are emerging mechanisms of diabetic‐linked PCOS [39]. Together, these interconnected genetic, hormonal, and environmental factors synergistically impair glucose homeostasis in women with PCOS, significantly elevating their risk of developing DM.

Given this elevated risk, timely prevention, early screening, and accurate detection of impaired glucose tolerance are critically important in women with PCOS to reduce progression to overt DM and to implement appropriate therapeutic interventions aimed at mitigating long‐term metabolic complications.

The strength of this study lies in its application of a multi‐state model, which enables us to account for the often‐neglected intermediate stage of pre‐DM in binary models and assess the effect of PCOS on transitions. Moreover, the dataset included longitudinal follow‐up, which may capture lifetime risks of DM. The comprehensive data collection within the TLGS, which included standardized anthropometric assessments, ultrasound evaluations, and fasting blood samples collected during the early follicular phase, ensured reliable and consistent measurement of PCOS diagnostic criteria. Moreover, the clear delineation of study groups, comprising women diagnosed with PCOS according to established criteria, facilitated a precise comparison among distinct groups. This rigorous approach enabled the generation of novel insights into the spectrum of metabolic and clinical transitions associated with PCOS, thereby enhancing the understanding of its dynamic progression over time. Despite the strength of this study, several limitations should be acknowledged. Although we evaluated risk factors influencing transitions in glycemic states, there are also additional factors, such as genetic predisposition, lifestyle behaviours, and comorbid conditions, which may affect the progression of blood glucose impairment and were not assessed in our analysis. Also, the multi‐state model was specified to capture the first progression through the sequence of normoglycemia, pre‐DM, and DM; therefore, only the first entry into each state was considered. Consequently, reverse transitions, such as regression from pre‐DM to normoglycemia, and subsequent recurrent transitions were not modelled. Because glycemic status may fluctuate over time, this approach may not fully capture the dynamic and potentially reversible nature of glucose dysregulation. Future studies using recurrent or more flexible multi‐state models that allow bidirectional and repeated transitions may provide a more comprehensive representation of the longitudinal evolution of glycemic states. Other limitations of this study are the exclusion of women presenting with only isolated features of PCOS who did not meet the full Rotterdam criteria. While this exclusion was necessary to ensure the reliability of our classification and to avoid diagnostic ambiguity, we recognize that it may limit the generalizability of our findings. Moreover, we treated PCOS status as a baseline exposure rather than a time‐varying one. Another limitation of this study is the absence of HbA1c measurements. Relying exclusively on FPG and 2‐h post‐load glucose may have resulted in the under‐detection of certain cases of pre‐DM and DM that present primarily with elevated HbA1c levels but normal glucose tolerance. Furthermore, we were unable to examine the impact of different PCOS treatment modalities, including pharmacological and lifestyle interventions, on the transition probabilities due to data limitations. Further well‐designed longitudinal cohort studies with frequent and comprehensive metabolic assessments are required to validate and extend our findings.

## 5 Conclusions

Women with PCOS had a significantly higher hazard of transitioning from normoglycemia to pre‐DM compared with women without PCOS, whereas PCOS was not significantly associated with direct progression from normoglycemia to DM or progression from pre‐DM to DM. These findings highlight the importance of early identification and monitoring of dysglycemia, particularly the transition from normoglycemia to pre‐DM, among women with PCOS.

## Author Contributions

**Fahimeh Ramezani Tehrani:** conceptualization, investigation, writing – original draft, writing – review and editing, visualization, validation, methodology, project administration, supervision. **Fereidoun Azizi:** investigation, conceptualization, methodology, writing – review and editing, writing – original draft. **Maryam Mousavi:** methodology, investigation, writing – original draft, writing – review and editing, formal analysis, software, data curation, visualization. **Mahsa Noroozzadeh:** writing – original draft, writing – review and editing, investigation. **Marzieh Saei Ghare Naz:** investigation, writing – original draft, writing – review and editing, conceptualization, methodology.

## Funding

This study was supported by the Elite Researcher Grant Committee under award number IR.NIMAD.REC.4030671 from the National Institute for Medical Research Development, Tehran, Iran.

## Ethics Statement

The study protocol complies with the Declaration of Helsinki and was approved by the Research Ethics Committees of National Institute for Medical Research Development (Ethics code: IR.NIMAD.REC.1404.010).

## Consent

Informed consent was obtained from all participants.

## Conflicts of Interest

The authors declare no conflicts of interest.

## Data Availability

The data that support the findings of this study are available on request from the corresponding author. The data are not publicly available due to privacy or ethical restrictions.
